# Whole genome sequencing data of 14 indigenous Greek goats

**DOI:** 10.1038/s41597-025-05999-2

**Published:** 2025-10-30

**Authors:** Antiopi Tsoureki, Sofia Michailidou, Sotiria Vouraki, Evridiki Boukouvala, Georgios Arsenos, Ioannis Sakaridis

**Affiliations:** 1 Institute of Applied Biosciences, Centre for Research and Technology Hellas, 57001 Thessaloniki, Greece; 2 Laboratory of Animal Production, Nutrition and Biotechnology, Department of Agriculture, School of Agriculture, University of Ioannina, 47100 Arta, Greece; 3 Laboratory of Animal Husbandry, School of Veterinary Medicine, Faculty of Health Sciences, Aristotle University of Thessaloniki, 54124 Thessaloniki, Greece; 4 Hellenic Agricultural Organization DIMITRA, Campus of Thermi, 57001 Thessaloniki, Greece

**Keywords:** Next-generation sequencing, Agricultural genetics, Genetic variation

## Abstract

Goat farming is a significant livestock sector in Greece, which holds the largest population of goats in the European Union. This population is mainly composed of the Eghoria and Skopelos indigenous breeds, the first of which is characterized by great phenotypic diversity, while the second presents a more uniform phenotype. Both breeds are characterized by high levels of genetic diversity. However, data regarding their genetic structure are scarce, usually concerning a limited number of genetic loci. Here, we present the first whole genome sequencing data generated for 14 indigenous Greek goats. In total, 66.5 Gb of data were produced on a NovaSeq. 6000 Illumina sequencer, corresponding to 3.18X average coverage. After quality filtering, >99.7% of sequences mapped successfully to the goat reference genome. Variant calling identified approximately 14 million autosomal variants of high-quality. These data can be used for the genetic improvement of the national herd through selective breeding schemes and, subsequently, improve the sustainability of the sector.

## Background & Summary

Goat farming is a significant agricultural activity in Greece with vast socioeconomic and environmental impact^1^. The national herd is the largest in EU, comprising 2.58 million individuals in 2024, with Greece constituting one of the main goat milk producers in the EU^2^. Despite the large number of reared goats in Greece, the overall milk production is comparatively moderate, indicating the potential of the Greek goat population for genetic improvement.

Greek goat populations are represented mainly by two breeds, namely Eghoria and Skopelos. Eghoria breed includes approximately 90% of all individuals and it has a nationwide distribution. Skopelos breed constitutes less than the remaining 10% of the total population (the rest belonging to various foreign breeds and their crosses with indigenous ones) and its distribution is limited mainly to the Northern Sporades Island complex, with some populations reared in other parts of Greece. These breeds are primarily reared for their milk, which is used for the production of various traditional dairy products, many of which are of Protected Designation of Origin (PDO) and Protected Geographical Indication (PGI). Phenotypically, Eghoria breed displays a high degree of variability in terms of coat color (black, brown, white or combinations of them), it has long hair, and produces 100–250 kg of milk per milking period. Skopelos breed is characterized by great homogeneity, with brown hair of short length, and 200–400 kg milk yield per milking period. Both breeds are able to efficiently utilize poor pastures and are well adapted to dry and hot climatic conditions^3^. In particular, a recent study identified Runs of Homozygosity (ROHs) harbouring genes linked to heat stress response and heat resilience in both breeds, confirming their potential for adaptation to local semi-arid and hot-arid environments. Additionally, ROHs encompassing immune-related genes were also detected in both breeds, suggesting the existence of resilience to endemic diseases linked to local disease-related challenges^4^. Genetically, both breeds present high levels of variation^4,5^, indicating high potential for genetic improvement^6^.

Genetic studies of Greek goat breeds are limited, with the majority of them focusing on the Skopelos breed, while Eghoria breed is largely understudied^6^. Most of these studies examine a small number of genetic loci and their correlation with specific traits^7–12^. In terms of population genetics, the number of studies is even smaller, concerning either a limited number^13,14^ or Single Nucleotide Polymorphism (SNP) microarrays^4,5^. Although genotyping microarrays remain a cost-effective and widely used technology for genomic analyses, whole genome sequencing (WGS) is expected to become the method of choice in the following years as sequencing costs keep decreasing^15^. Moreover, although the goat genome is publicly available since 2012^16^, no WGS data have been generated so far for the Greek goat breeds.

**Fig. 1:**
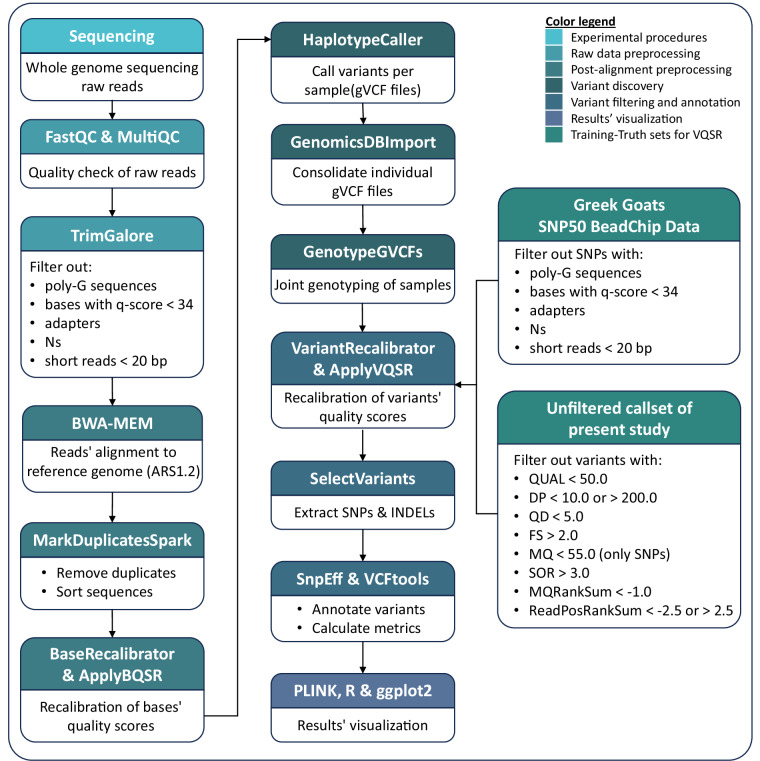
Overview of the workflow followed during bioinformatic analysis of the goats’ whole genome sequencing (WGS) data. Different stages of the analysis are denoted by different colors.

Here, we report the first WGS data of 14 indigenous Greek goats (*Capra hircus*) from six populations, belonging to the Eghoria and Skopelos breeds, along with the methods implemented to acquire the final callset from the raw data (Fig. 1). The data include approximately 14 million variants (SNPs and Insertions and Deletions - INDELs) of high quality. These data constitute the beginning of a nationwide database containing information on the genetic background of Greek goats. Such a database can be utilized for the comprehensive genetic characterization of Greek goat populations and the elucidation of their potential for improvement. In addition, these data can be used for breed and product traceability as well as the identification of genetic loci correlated with important traits such as milk and meat production, disease resistance, and resilience or adaptability to environmental changes. Altogether, the data presented here can help in designing targeted breeding schemes and informed conservation strategies, contributing to the overall sustainability of Greek goat husbandry.

## Methods

### Sampling and DNA extraction

**Fig. 2:**
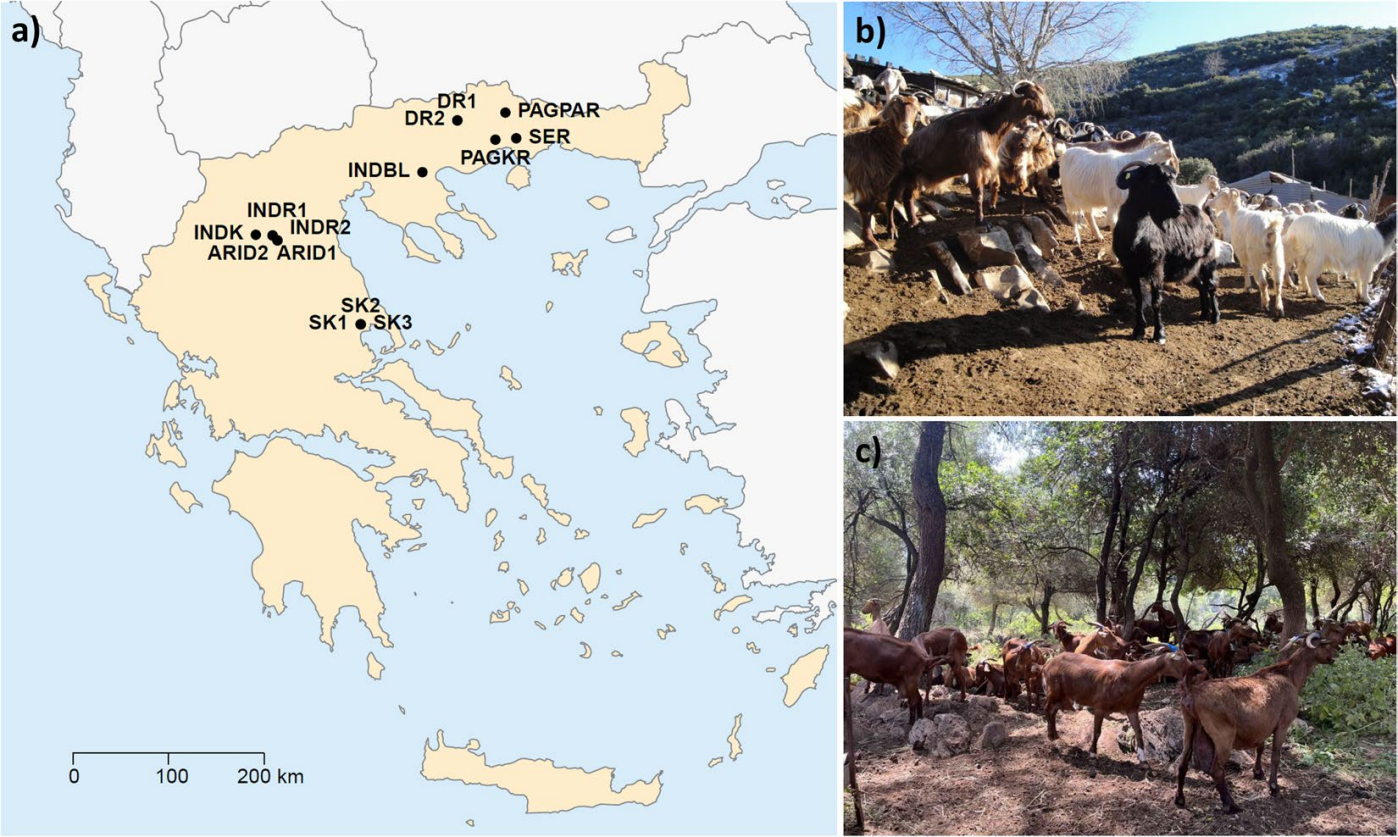
Farm locations and phenotypes of the populations and breeds included in the study. (**a**) Map of Greece indicating the locations of the farms from which samples were collected, (**b**) representative individuals of the Eghoria and (**c**) Skopelos breeds sampled within this study. Population abbreviations: ARID: Aridaia, DR: Drama, IND: Indigenous, PAG: Paggaio, SER: Serres, SK: Skopelos.

**Fig. 3:**
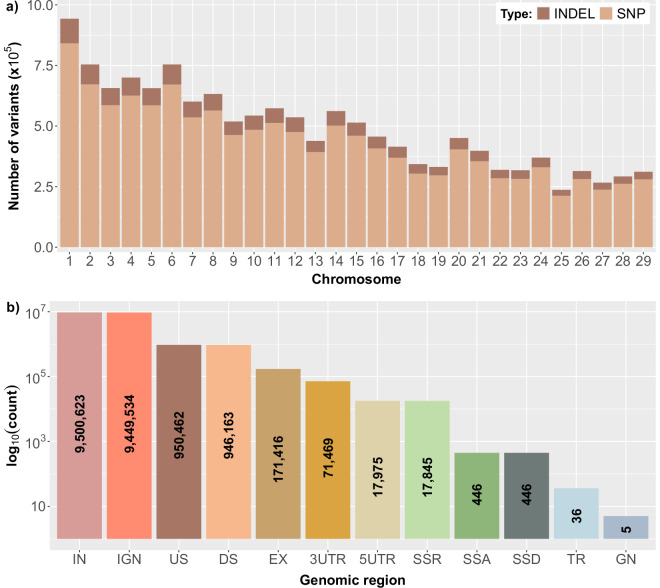
Distribution of variants per chromosome and category of genomic region. (**a**) Total number of variants detected in each autosomal chromosome. The type of the variants (SNPs or INDELs) is denoted by different colors (light brown: SNPs, dark brown: INDELs), (**b**) Number of variants detected in the different categories of genomic regions. The number displayed inside each bar represents the number of variants identified in each genomic region category. IN: intron, IGN: intergenic, US: upstream, DS: downstream, EX: exon, 3UTR: 3′ untranslated region, 5UTR: 5′ untranslated region, SSR: splice site region, SSA: splice site acceptor, SSD: splice site donor, TR: transcript, GN: gene.

Breeds from 9 different farms in Northern and Central Greece (Fig. 2a), were studied, comprising a total of 14 goats: 11 from the Eghoria (Fig. 2b) and 3 from the Skopelos (Fig. 2c) breed. Due to the high morphological and phenotypic variation of the Eghoria breed, five distinct populations of this breed were included in the analysis, in order to capture most of its genetic diversity (Table 1). Goats were selected as purebred representatives of the above breeds according to their morphological characteristics. Individual blood samples were collected from the jugular vein in tubes containing EDTA as anticoagulant and stored in a freezer (−20 °C) until further laboratory use. DNA extraction was performed using the kit PureLink^TM^ Genomic DNA kit (Thermo Fisher Scientific, Waltham, MA, USA) according to the manufacturer’s instructions. Isolated DNA was quantified using the Eppendorf μCuvette® G 1.0 and Eppendorf BioSpectrometer (Eppendorf, Hamburg, Germany) and its quality and integrity was assessed with agarose (0.7%) gel electrophoresis. The required amount of DNA (1 μg) was shipped to Macrogen Inc. (Amsterdam, The Netherlands, https://www.macrogen-europe.com/) for sequencing according to the company’s requirements.

**Table 1:** List of samples collected for whole genome sequencing and their information about breed, population, and farm location.

Sample ID	Breed	Population	Farm location
ARID1	Eghoria	Aridaia	Aiani, Kozani
ARID2	Eghoria	Aridaia	Aiani, Kozani
DR1	Eghoria	Drama	Prosotsani, Drama
DR2	Eghoria	Drama	Prosotsani, Drama
INDBL	Eghoria	Indigenous	Lefkouda, Thessaloniki
INDK	Eghoria	Indigenous	Kozani-Grevena
INDR1	Eghoria	Indigenous	Rimnio, Kozani
INDR2	Eghoria	Indigenous	Rimnio, Kozani
PAGKR	Eghoria	Paggaio	Krioneri, Drama
PAGPAR	Eghoria	Paggaio	Paranesti, Drama
SER	Eghoria	Serres	Makrihori, Kavala
SK1	Skopelos	Skopelos	Sesklo, Magnisia
SK2	Skopelos	Skopelos	Sesklo, Magnisia
SK3	Skopelos	Skopelos	Sesklo, Magnisia

### Library preparation and sequencing

Libraries were constructed with the TruSeq DNA PCR-Free kit (Illumina Inc., San Diego, CA, USA) following Illumina’s protocol “TruSeq DNA PCR-Free Sample Preparation Guide, Part #15036187 Rev. D”. Libraries were sequenced on an Illumina NovaSeq. 6000 platform using the S4 Reagent Kit v1.5 (300 cycles) (Illumina Inc., San Diego, CA, USA) resulting in the production of raw paired-end 150 bp sequences for each sample.

### Sequence alignment and variant discovery

Raw sequences’ quality was checked using FastQC (v.0.11.7)^17^ and MultiQC (v.1.11)^18^. Subsequently, trimming was performed using TrimGalore (v.0.6.7)^19^ with the “–2colour” option, to remove poly-G sequences, lower quality bases (q-score < 34), adapter sequences, unidentified nucleotides (N), and very short sequences (<20 bases) from the data. Trimmed reads were aligned to the *Capra hircus* reference genome ARS1.2 (GCA_001704415.2) with the Burrows-Wheeler Aligner (version 0.7.17-r1188) using the BWA-MEM algorithm^20^. For variant discovery, the Genome Analysis Toolkit (GATK, v.4.1.8.1)^21^ was employed. Specifically, duplicate sequences were removed and the remaining were sorted with the “MarkDuplicatesSpark” function, a plug-in implementation of Picard’s “MarkDuplicates”^22^. Then, Base Quality Score Recalibration was performed on the data to correct bases’ quality score for systematic technical errors. GATK’s HaplotypeCaller^23^ was employed to calculate the genotype likelihoods for each sample and produce individual gVCF files. The individual gVCF files were consolidated with the “GenomicsDBImport” tool and joint genotyping of the samples followed, using the “GenotypeGVCFs” tool, resulting in a single VCF file containing the raw SNPs and INDELs.

### Variant filtering

After obtaining the genotypes for all samples, variant quality score recalibration (VQSR) was conducted to filter out low-quality variants. The model for VQSR was built with the “VariantRecalibrator” tool, using two custom training and truth resource sets. The first custom set was generated in a previous study^4^, in which 72 animals belonging to the two Greek goat breeds (32 to Eghoria and 40 to Skopelos breed) were genotyped with Illumina’s Goat SNP50 BeadChip^24^. Raw SNPs were filtered based on MAF (<1%), call rate (<0.98), and Hardy-Weinberg equilibrium (HWE p-value ≤ 1.0E-6) as well as genomic location (SNPs that lacked genomic location or were located on sex chromosomes were excluded) as described in Michailidou *et al*. (2019), resulting in a total of 48,841 high-quality SNPs capable of capturing the genetic variation of Greek goat populations. This set of 48,841 SNPs was used as training and truth set.

For the generation of the second custom set, the highest-confidence variants were obtained from our callset by hard-filtering the raw variants using stringent thresholds. In particular, the SNPs’ exclusion criteria were at least one of the QUAL < 50.0, DP < 10.0, DP > 200.0, QD < 5.0, FS > 2.0, MQ < 55.0, SOR > 3.0, MQRankSum < −1.0, ReadPosRankSum < −2.5 or ReadPosRankSum > 2.5, while for INDELs the same filters were applied with the exception of MQ < 55.0. The resulting set, consisting of 10,846,918 high-confidence variants, was used as training and truth set. The known variants available for the goat reference genome at Ensembl version 112^25^ were used as the known resource set. SNPs and INDELs below the 99.0% sensitivity threshold were removed from the dataset. Further filtering was applied to remove variants with a depth across all samples greater than 110X, monomorphic and multiallelic variants, as well as INDELs longer than 50 bases.

### Annotation and visualization

Variants’ annotation was performed with SnpEff (v.5.2c)^26^. For the evaluation of variants’ quality, the Ti/Tv ratio was examined and mean variant depth and SNP density in 1 Kilobase (Kb) windows were calculated with VCFtools (v.0.1.16)^27^.

Population structure was examined by Principal Component Analysis (PCA). For PCA, the final variant callset was further filtered to obtain a thinned set of high-quality variants. Specifically, variants with MAF < 0.05 as well as those that were not called in more than 4 samples were filtered out. The remaining variants were thinned by selecting one variant per 50 kb. The remaining 48,809 variants were then used for PCA. PCA was performed using PLINK v1.9^28^. All statistics and data visualizations were performed in R programming language (v.4.1.0)^29^ using the ggplot2 package (v.3.4.2)^30^.

## Data Records

The raw whole genome sequencing data in fastq format from the 14 indigenous Greek goats belonging to 6 populations have been deposited to NCBI’s Sequence Read Archive (SRA) repository and are accessible under the accession number PRJNA1173400^31^. The final variant callset has been deposited to the European Nucleotide Archive (ENA) at EMBL-EBI, under the accession number PRJEB95944^32^.

## Technical Validation

### Sequence quality

After sequencing, 9.21 Gigabases (Gb) of data were produced on average for all samples, ranging from 7.32 to 13.02 Gb per sample (Table 2). This corresponded to an average genome coverage of 3.18X per sample, ranging from 2.52X to 4.49X. The percentage of high-quality bases with a minimum Phred scaled quality score of 30 equaled 90.45% on average for the raw data, with a range from 88.45% to 91.5% for each sample. After trimming and quality filtering, the average percentage increased to 92.82%, ranging from 91.74% to 93.49% for the individual samples.

**Table 2:** Sequencing and alignment metrics for each sample.

Sample ID	Coverage (X)	Total Gigabases (Gb)	Raw Sequences	Trimmed sequences	Trimmed sequences’ length (bases)	Alignment rate (%)
ARID1	2.68	7.79	51,561,006	47,194,890	143	99.78
ARID2	2.67	7.73	51,198,964	47,191,124	144	99.83
DR1	4.49	13.02	86,238,690	82,509,572	146	99.74
DR2	3.06	8.89	58,847,412	56,450,222	146	99.83
INDBL	3.22	9.33	61,770,066	57,735,740	145	99.85
INDK	3.44	9.98	66,116,164	62,750,176	145	99.82
INDR1	2.87	8.31	55,024,014	52,329,632	145	99.81
INDR2	3.75	10.88	72,041,856	68,916,150	146	99.79
PAGKR	3.07	8.89	58,896,036	56,281,150	146	99.85
PAGPAR	3.73	10.82	71,645,864	67,349,194	145	99.79
SER	2.78	8.06	53,368,654	50,989,412	146	99.87
SK1	3.55	10.29	68,146,926	65,461,350	146	99.82
SK2	2.63	7.62	50,484,704	47,740,952	146	99.82
SK3	2.52	7.32	48,484,258	45,411,270	145	99.81

The appearance of poly-G sequences in the data is a known issue on 2-color systems, such as the NovaSeq 6000 sequencing platform, used in the present study. Specifically, in 2-color systems, adenine (A) produces signal in both channels, cytosine (C) and thymine (T) produce signal in either channel, and guanine (G) is unlabeled. However, the sequencer cannot distinguish if the absence of signal is due to a G base or issues encountered during sequencing, resulting in overcalling of high-quality G bases in the reads^33–35^. Consequently, the Phred scaled quality score-based filtering is rendered ineffective in this case. Therefore, in order to eliminate these artificial poly-G sequences, the appropriate indication that the data were generated on a 2-color system is required at the quality filtering step. This specification directs the algorithm to ignore quality scores of G bases during read trimming, thus effectively removing the false poly-G sequences.

In the present study, this filtering approach, along with the rest of the filtering criteria applied, resulted in the reduction of the average number of reads per sample from 60,987,472 (range from 48,484,258 to 86,238,690) to 57,736,488 (range from 45,411,270 to 82,509,572), while the respective average length of the reads per sample was reduced from 151 bases to 145 bases (range from 140 to 147). After quality filtering, the alignment rate achieved exceeded 99.7% for all samples (Table 2).

### Variants’ quality

In total, 18,470,503 raw autosomal variants were identified. Variant recalibration and subsequent filtering at the 99.0% sensitivity threshold resulted in the exclusion of 3,904,471 variants from the data. The remaining variants were further filtered based on their mean coverage across all samples, with those exceeding the mean + 6*SD coverage value (equal to 110X) being excluded from the callset, as they constitute artifacts arising during alignment^36^. Along with the exclusion of monomorphic and multiallelic variants, and INDELs longer than 50 bp, the final callset consisted of 14,200,959 high-quality variants. Of these, 12,670,446 were SNPs, 691,134 were insertions, and 839,379 were deletions. Among the final high-quality variants, 13,753,517 were successfully genotyped in at least half the samples included in the study (variant missingness < 0.5), while 838,877 were genotyped in all 14 samples. The number of genotyped, polymorphic variants in each sample ranged from 2,884,133 (2,623,734 SNPs and 260,399 INDELs) in sample SK3 to 4,360,057 (3,942,461 SNPs and 417,596 INDELs) in sample DR1, while missingness ranged from 0.286 to 0.135, respectively, for the same samples (Table 3).

**Table 3:** Number of genotyped, polymorphic variants (total variants, SNPs only, and INDELs only), missingness, and Transition to Transversion (Ti/Tv) ratio for each sample.

Sample ID	No. of total variants	No. of SNPs	No. of INDELs	Missingness	Ti/Tv ratio
ARID1	2,970,298	2,700,904	269,394	0.270	2.373
ARID2	3,003,428	2,728,220	275,208	0.267	2.369
DR1	4,360,057	3,942,461	417,596	0.135	2.363
DR2	3,278,208	2,971,758	306,450	0.233	2.367
INDBL	3,249,256	2,950,535	298,721	0.241	2.373
INDK	3,600,551	3,264,215	336,336	0.208	2.370
INDR1	3,007,885	2,731,465	276,420	0.272	2.370
INDR2	3,845,171	3,482,153	363,018	0.180	2.370
PAGKR	3,284,197	2,977,532	306,665	0.241	2.370
PAGPAR	3,779,287	3,425,218	354,069	0.189	2.369
SER	3,329,507	3,019,547	309,960	0.235	2.369
SK1	3,774,549	3,416,949	357,600	0.185	2.368
SK2	3,086,853	2,804,302	282,551	0.262	2.375
SK3	2,884,133	2,623,734	260,399	0.286	2.388

The total number of variants detected in each autosomal chromosome was mildly correlated with the chromosome’s length, which was also true when SNPs and INDELs were examined separately (Fig. 3a). Moreover, the SNPs to INDELs ratio was relatively consistent across the chromosomes (8.30 ± 0.27), indicating the homogenous distribution of each variant type across the goat genome (Table 4).

**Table 4:** Variant metrics per chromosome.

Chromosome	No. of variants	No. of SNPs	No. of INDELs	SNPs/INDELs ratio	SNP density (per 1 Kb) (mean ± s.d.)
**1**	942,849	840,464	102,385	8.21	5.99 (±4.40)
**2**	753,934	671,874	82,060	8.19	5.52 (±4.11)
**3**	656,413	586,095	70,318	8.33	5.47 (±4.46)
**4**	699,736	625,742	73,994	8.46	5.80 (±4.32)
**5**	656,035	585,707	70,328	8.33	5.51 (±4.28)
**6**	753,888	671,522	82,366	8.15	6.41 (±4.68)
**7**	600,708	535,571	65,137	8.22	5.54 (±4.58)
**8**	632,261	563,636	68,625	8.21	5.61 (±4.22)
**9**	518,743	462,427	56,316	8.21	5.67 (±4.19)
**10**	543,164	483,863	59,301	8.16	5.38 (±4.37)
**11**	573,157	512,569	60,588	8.46	5.40 (±4.02)
**12**	536,151	475,328	60,823	7.81	6.14 (±4.93)
**13**	438,431	392,648	45,783	8.58	5.28 (±3.96)
**14**	561,621	501,526	60,095	8.35	5.93 (±4.47)
**15**	513,896	459,395	54,501	8.43	6.27 (±5.00)
**16**	456,317	407,596	48,721	8.37	5.75 (±4.50)
**17**	414,389	368,711	45,678	8.07	5.83 (±4.40)
**18**	342,904	303,382	39,522	7.68	5.10 (±4.16)
**19**	331,235	296,114	35,121	8.43	5.30 (±4.59)
**20**	450,680	403,492	47,188	8.55	6.28 (±4.49)
**21**	397,578	354,728	42,850	8.28	5.73 (±4.63)
**22**	319,262	283,946	35,316	8.04	5.30 (±4.00)
**23**	317,414	281,867	35,547	7.93	6.50 (±6.63)
**24**	369,645	329,861	39,784	8.29	5.93 (±4.34)
**25**	236,677	212,483	24,194	8.78	5.52 (±4.13)
**26**	314,321	280,902	33,419	8.41	6.11 (±4.66)
**27**	266,425	237,269	29,156	8.14	5.96 (±4.46)
**28**	291,774	261,944	29,830	8.78	6.53 (±4.88)
**29**	311,351	279,784	31,567	8.86	6.07 (±4.61)

Number of total variants, number of SNPs, number of INDELs, SNPs/INDELs ratio, and SNP density per autosomal chromosome.

Variant annotation yielded 21,126,420 annotations for the final callset, as most variants were assigned to multiple types of genomic regions. The vast majority of the variants were located in non-coding regions. In particular, 44.97% and 44.73% were located in intronic and intergenic regions, respectively, while 4.50% and 4.48% were located in areas upstream and downstream of genes, respectively. On the contrary, only 0.81% of the total variants were detected in exons (Fig. 3b).

**Fig. 4:**
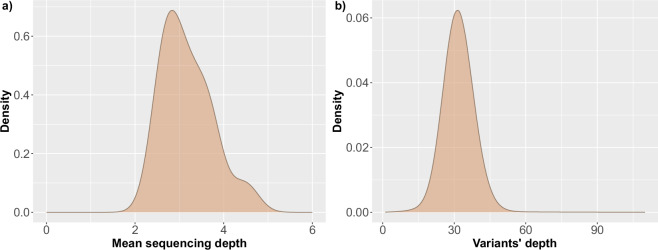
Sequencing quality metrics for the data. (**a**) Density plot showing the distribution of mean sequencing depth achieved for the 14 samples, (**b**) Density plot showing the distribution of variants’ depth for the entire callset.

Variant quality was assessed through their mean depth, while for SNPs specifically the Transition to Transversion (Ti/Tv) ratio and the SNP density were also examined. Low coverage sequencing presents a challenge during identification of variant sites and genotyping, due to the limited amount of data available for any given site in each individual sequenced sample. Joint genotyping addresses this by combining the available data from all samples in a dataset, to detect the variant sites in each individual sample with a high level of sensitivity^37^. Thus, despite the low coverage achieved during sequencing for the individual samples in the current study (mean = 3.18X, s.d. = 0.56) (Fig. 4a), the application of joint genotyping allowed for calling variants with increased sensitivity. Specifically, by aggregating the total number of reads across all samples at the genotyping step of the analysis, an average depth of 31.87X (s.d. = 7.04) was achieved for the identified variants (Fig. 4b). Filtering of the callset based on minimum variant depth revealed that 75.94% (10,783,825) of the variants had a minimum depth of 28 sequences, while only 7.73% (1,097,953) of them had a minimum depth of 42 sequences across all samples, corresponding, approximately, to 2 and 3 sequences per sample on average, respectively. This result highlights the major benefit of employing joint genotyping for low-coverage samples.

The Ti/Tv ratio, which is an indicator of the overall SNP quality^38^, in the initial raw callset was equal to 2.33, which further increased, after variant filtering, to 2.37 for the final callset, indicating good quality of the SNP calling. For the individual samples, the average Ti/Tv ratio equaled 2.371 (s.d. = 0.006), ranging from 2.363 in sample DR1 to 2.388 in sample SK3 (Table 3).

In addition, SNP density in the final callset ranged from 5.10 (s.d. = 4.16) to 6.53 (s.d. = 4.88) per Kb in chromosomes 18 and 28, respectively (Table 4). These values are quite lower than the 1 variant per 10 bp suggested threshold indicating the presence of false positive calls in the data^38^, highlighting the high confidence of the SNPs included in the final callset.

**Fig. 5:**
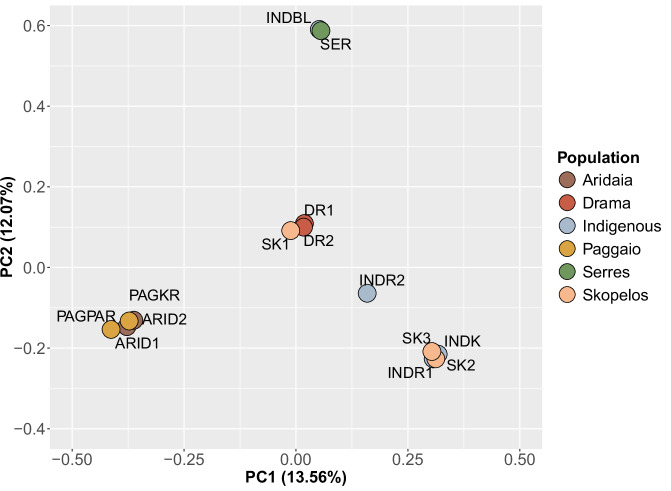
Principal Component Analysis for the Greek goat samples included in the study. Different populations are denoted by different colors.

PCA analysis revealed the genetic relationship of the six goat populations. In particular, PCA showed that there is no clear breed or population distinction for the samples included in the study (Fig. 5). This finding aligns with a previous study on Greek goat breeds, in which the close genetic relatedness between the Eghoria and Skopelos breeds was confirmed^5^. However, these breeds have distinct ROH patterns, which reflect the different management practices and selection pressure applied for mainland and insular breeds^4^. The high degree of genetic variation in the Greek goat populations confirms the absence of coordinated breeding schemes, especially for the Eghoria breed. Such schemes could exploit and manage the available genetic resources, in order to guide the selection strategies applied by farmers, with the aim of improving individuals’ phenotypic characteristics and performance traits. Consequently, the need for structured, targeted breeding programs incorporating such genetic information and for the application of conservation policies for the Greek goats is highlighted.

## Data Availability

The raw WGS data are available at the Sequence Read Archive (SRA, NCBI) repository under the accession number PRJNA1173400^31^, while the variation data are available at the European Nucleotide Archive (ENA, EMBL-EBI), under the accession number PRJEB95944^32^.

## Code availability

The workflow and commands used for the analysis of the current dataset are available in https://github.com/atsoureki/Variant_Calling_Goats^39^.
